# Supplementary material for: Evidence for immune activation in pathogenesis of the HLA class II associated disease, podoconiosis
Source: Nat Commun. 2024 Mar 6;15:2020. doi: 10.1038/s41467-024-46347-z (PMC10917762; doi:10.1038/s41467-024-46347-z)
Supplement: Supplementary file 1 — Supplementary Information [file 41467_2024_46347_MOESM1_ESM.pdf]

Evidence for immune activation in pathogenesis of the HLA class II associated disease, podoconiosis

***Mikias Negash[1,2,3], Menberework Chanyalew[2], Tigist Girma[2], Fekadu Alemu[2], Diana Alcantara[1], Ben Towler[4], Gail Davey[1,7], Rosemary J Boyton[5], Daniel M Altmann[6], Rawleigh Howe[2] and Melanie J. Newport[1]**

[1]Brighton and Sussex Centre for Global Health Research, Department of Global Health and Infection, Brighton and Sussex Medical School, Brighton, UK; [2]Armauer Hansen Research Institute, Addis Ababa, Ethiopia; [3]Department of Medical Laboratory Science, College of Health Sciences, Addis Ababa University, Ethiopia; [4]Department of Biochemistry and Biomedicine, School of Life Sciences, University of Sussex; [5]Department of Infectious Disease, Imperial College London; [6]Department of Immunology and Inflammation, Imperial College London. [7]School of Public Health, Addis Ababa University, Ethiopia

*Corresponding author: mikiasn2@gmail.com

Supplementary tables and figures

Supplementary table 1. Socio-demographic characteristics of podoconiosis patients and healthy controls in North West Ethiopia.

Variable	Podoconiosis n = 64	HC (%), n = 49
**Sex:** Male	36 (55.7)	26 (53.1)
Female	28 (44.3)	23 (46.9)
**Age (years):** Mean (min-max)	47.8 (28-72)	34.4 (21-60)
**Occupation:** Farmer<br>Student<br>Trader	64 (100)	43 (87.8)<br>5 (10.2)<br>1 (2)
**Marital status:** Married<br>Divorced<br>Single	53 (82.8)<br>8 (12.5)<br>3 (4.7)	37 (75.5)<br>5 (10.2)<br>7 (14.3)
**Disease stage:** Stage 2<br>Stage 3	59 (92.2)<br>5 (7.8)	
**Duration of disease in years:** mean (sd)	19 (11.2)	
**Site involved:** Both legs<br>Left leg<br>Right leg	58 (90.6)<br>4 (6.3)<br>2 (3.1)	

Supplementary table 2. Number and type of matched samples for RNA-Seq.

**Variables**	**Podo patients** mean(sd)	**Healthy controls** mean(sd)
Age:	41.3 (8.1)	34 (3.9)
PBMC count $x10^6$	7.6 (3.1)	8.1 (3.8)
RNA yield ng/µl	41 (19.6)	42 (20.7)
Sex: Male	11	12
Female	13	12
Total	24	24

Supplementary table 3. Number of reads mapped to the human reference genome GRCh38 using featureCounts in peripheral blood from 23 healthy control samples

Sample	Total no of input reads	Reads with adapter	Reads passing filter	Concordants (Aligned only 1 time)	Aligned > 1 time	Successfully assigned reads	Overall alignment rate
HC-14	33.832.817	1.408.022 (4.2%)	33,470,978 (98.9%)	29,191,614 (87.21%)	3,118,379 (9.32%)	23,955,289 (60.4%)	96.53%
HC-15	33,915,788	1,058,229 (3.1%)	33,841,422 (99.8%)	29,796717 (88.05%)	2,750712 (8.13%)	24,592985 (61.9%)	96.18%
HC-16	27,084,512	2,470,269 (9.1%)	25,387,497 (93.7%)	22,181274 (87.37%)	2,287238 (9.01%)	18,197433 (60.7%)	96.38%
HC-17	46,003,233	2,772,937 (6.0%)	44,497,289 (96.7%)	38,831270 (87.27%)	4,166542 (9.36%)	31,483022 (59.6%)	96.63%
HC-23	47,431,701	13,949,456 (29.4%)	34,542,069 (72.8%)	30,572011 (88.51%)	2,857419 (8.27%)	25,173695 (62.3%)	96.78%
HC-36	9,562,314	339,535 (3.6%)	9,473,740 (99.1%)	5,634950 (59.48%)	1,453882 (15.35%)	3,486127 (26.8%)	74.83%
HC-37	15,307,167	738,175 (4.8%)	15,007,965 (98.0%)	13,057059 (87.00%)	1,345717 (8.97%)	10,146321 (56.8%)	95.97%
HC-40	33,910,260	1,030,634 (3.0%)	33,817,745 (99.7%)	29,856020 (88.29%)	2,820149 (8.34%)	24,623845 (62.0%)	96.62%
HC-41	*4,325,928*	*160,258 (3.7%)*	*4,280,274 (98.9%)*	*3,705706 (86.58%)*	*412,539 (9.64%)*	*3,011364 (58.6%)*	*96.21%*
HC-43	*3,626,048*	*382,422 (10.5%)*	*3,327,545 (91.8%)*	*2,545472 (76.50%*	*374,500 (11.25%)*	*1,607812 (37.6%)*	*87.75%*
HC-45	*2,689,891*	*756,406 (28.1%)*	*1,985,389 (73.8%)*	*1,620922 (81.64%)*	*206,551 (10.40%)*	*1,019355 (40.8%)*	*92.05%*
HC-47	27,183,868	936,415 (3.4%)	26,981,799 (99.3%)	23,868681 (88.46%)	2,258084 (8.37%)	19,604777 (61.7%)	96.83%
HC-48	12,575,974	2,966,591 (23.6%)	9,872,955 (78.5%)	8,439668 (85.48%)	887,576 (8.99%)	6,842806 (57.8%)	94.47%
HC-50	22,063,554	2,659,902 (12.1%)	19,968,611 (90.5%)	17,508683 (87.68%)	1,858207 (9.31%)	14,360325 (60.7%)	96.99%
HC-51	*246,530*	26,067 (10.6%)	226,956 (92.1%)	199,194 (87.77%)	21197 (9.34%)	163,680 (60.8%)	97.11%
HC-52	*3,134*	283 (9.0%)	2,925 (93.3%)	2504 (85.61%)	314 (10.74%)	2060 (58.4%)	96.34%
HC-53	32,268,402	1,849,447 (5.7%)	31,179,766 (96.6%)	24,627395 (78.99%)	3,642563 (11.68%)	2,647148 (49.1%)	90.67%
HC-55	27,376,534	1,349,452 (4.9%)	26,706,286 (97.6%)	21,967289 (82.26%)	2,772888 (10.38%)	17,692407 (53.9%)	92.64%
HC-56	15,186,939	1,913,667 (12.6%)	13,609,102 (89.6%)	10,717216 (78.75%)	1,507730 (11.08%)	8,220946 (47.5%)	89.83%
HC-57	16,171,781	769,392 (4.8%)	15,831,923 (97.9%)	13,829029 (87.35%)	1,340719 (8.47%)	11,428285 (61.4%)	95.82%
HC-58	29,381,624	1,205,573 (4.1%)	28,967,509 (98.6%)	25,708691 (88.75%)	2,204901 (7.61%)	21,228483 (63.0%	96.36%
HC-59	21,010,212	907,606 (4.3%)	20,695,174 (98.5%)	18,027005 (87.11%)	1,809821 (8.75%)	14872243 (60.7%)	95.85%
HC-60	19,025,823	714,183 (3.8%)	18,870,549 (99.2%)	16,543300 (87.67%)	1,666240 (8.83%)	13,662849 (61.2%)	96.50%

Samples labelled in green are from sequencing run 1 and those in red are from run 2.

Supplementary table 4. Number of reads mapped to the human reference genome GRCh38 using featureCounts in peripheral blood from 21 podoconiosis patients

Sample	Total no of input reads	Reads with adapter	Reads passing filter	Concordants (Aligned only 1 times)	Aligned > 1 times	Successfully assigned reads	Overall alignment rate
Pod-12	11,414,493	970,819 (8.5%)	10,776,617 (94.4%)	9,443402 (87.63%)	9,39766 (8.72%)	7661722 (60.6%)	96.35%
Pod-16	10,853,869	910,161 (8.4%)	10,197,774 (94.0%)	8,408397 (82.45%)	1,196502 (11.73%	6,730632 (53.2%)	94.19%
Pod-23	45,657,552	3,926,708 (8.6%)	42,982,996 (94.1%)	37,328319 (86.84%)	3,886459 (9.04%)	30471785 (60.0%)	95.89%
Pod-39	18,328,618	780,747 (4.3%)	18,079,537 (98.6%)	16,074042 (88.91%)	1,479435 (8.18%)	13,207054 (62.7%)	97.09%
Pod-41	32,479,188	1,133,143 (3.5%)	32,288,076 (99.4%)	28,166390 (87.23%)	2,666998 (8.26%)	23,279632 (61.5%)	95.49%
Pod-44	6,971,017	352,824 (5.1%)	6,841,654 (98.1%)	5,908574 (86.36%)	647,069 (9.46%)	1,315198 (53.5%)	95.82%
Pod-45	26,998,653	1,156,691 (4.3%)	26,603,529 (98.5%)	23,666371 (88.96%)	2,081959 (7.83%)	19,369579 (62.5%)	96.79%
Pod-52	26,611,493	1,207,462 (4.5%)	26,130,061 (98.2%)	23,145298 (88.58%)	2,164175 (8.28%)	19,056436 (62.1%)	96.86%
Pod-55	17,259,947	759,374 (4.4%)	16,968,300 (98.3%)	15,060395 (88.76%)	1,400344 (8.25%)	12,473562 (62.9%)	97.01%
Pod-57	27,883,558	936,848 (3.4%)	27,721,179 (99.4%)	24,823829 (89.55%)	2,250086 (8.12%)	20,586125 (63.8%)	97.67%
Pod-59	21,599,472	870,104 (4.0%)	21,326,180 (98.7%)	18,957381 (88.89%)	1,738022 (8.15%)	15,611861 (62.6%)	97.04%
Pod-62	28,251,610	1,192,867 (4.2%)	27,790,502 (98.4%)	24,149676 (86.90%)	2,518338 (9.06%)	19,637221 (59.0%)	95.96%
Pod-63	*7,535*	754 (10.0%)	7,002 (92.9%)	5631 (80.42%)	721 (10.30%)	4430 (50.9%)	90.72%
Pod-68	21,814,402	3,113,390 (14.3%)	19,230,983 (88.2%)	16,450179 (85.54%)	1,735936 (9.03%)	13,440016 (58.5%)	94.57%
Pod-71	13,216,781	487,542 (3.7%)	13,075,320 (98.9%)	11,153777 (85.30%)	1,180826 (9.03%)	9,140052 (58.6%)	94.33%
Pod-73	16,254,477	633,120 (3.9%)	16,104,338 (99.1%)	14,280321 (88.67%)	1,357925 (8.43%)	11,792291 (62.8%)	97.11%
Pod-77	30,180,542	1,870,270 (6.2%)	29,153,861 (96.6%)	26,207174 (89.89%)	2,161521 (7.41%)	21685582 (64.4%)	97.31%
Pod-78	7,113,257	327,986 (4.6%)	6,978,802 (98.1%)	6,126998 (87.79%)	600,547 (8.61%)	4,975897 (60.0%)	96.40%
Pod-82	8,815,390	489,131 (5.5%)	8,550,388 (97.0%)	6,998516 (81.85%)	885,174 (10.35%)	5,630263 (53.2%)	92.20%
Pod-86	29,062,114	1,741,053 (6.0%)	28,174,148 (96.9%)	25,149258 (89.26%)	2,304682 (8.18%)	20,644021 (63.1%)	97.44%
Pod-91	25,129,377	1,693,697 (6.7%)	24,146,243 (96.1%)	21,164493 (87.65%)	1,965354 (8.14%)	17,478451 (61.6%)	95.79%

Samples labelled in green are from sequencing run 1 and those in red are from run 2.

Supplementary figure 1

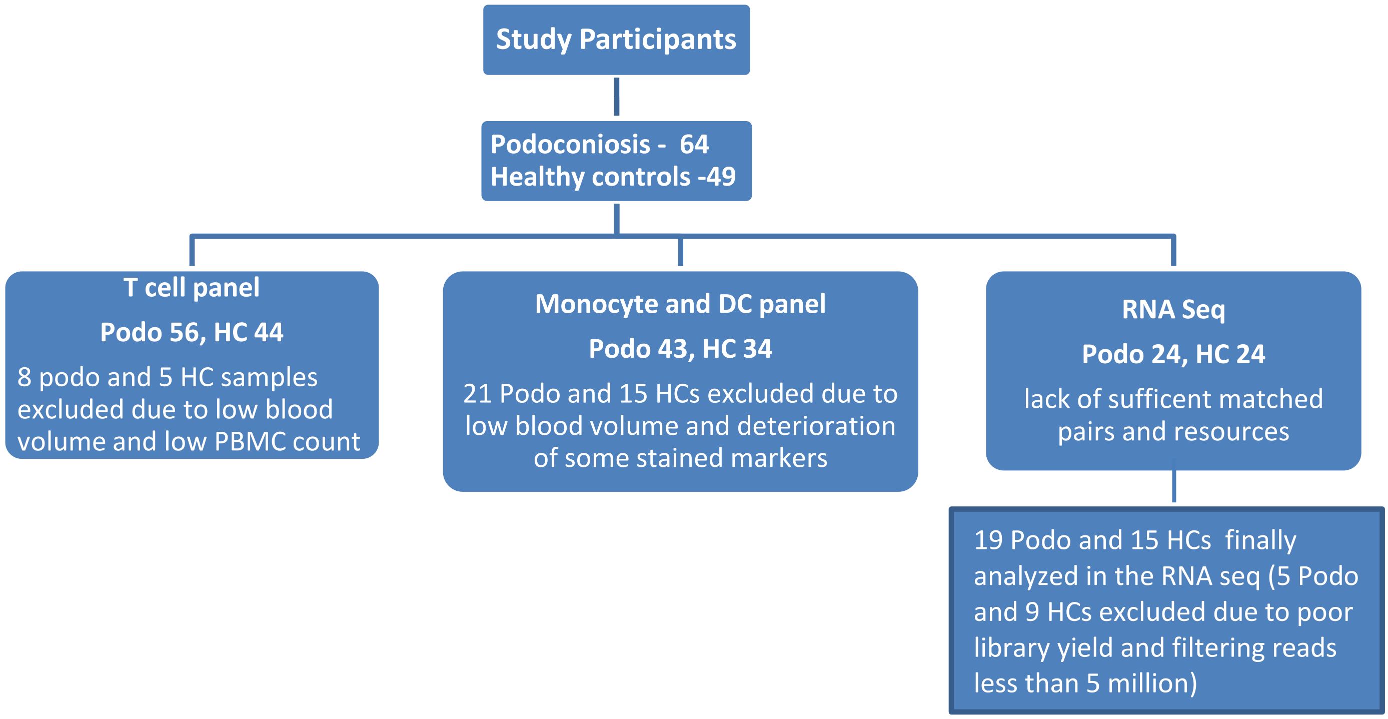

Supplementary Fig1. Flow chart on the number of samples analysed in the different panels and tests with their respective reason of exclusion.

Supplementary figure 2

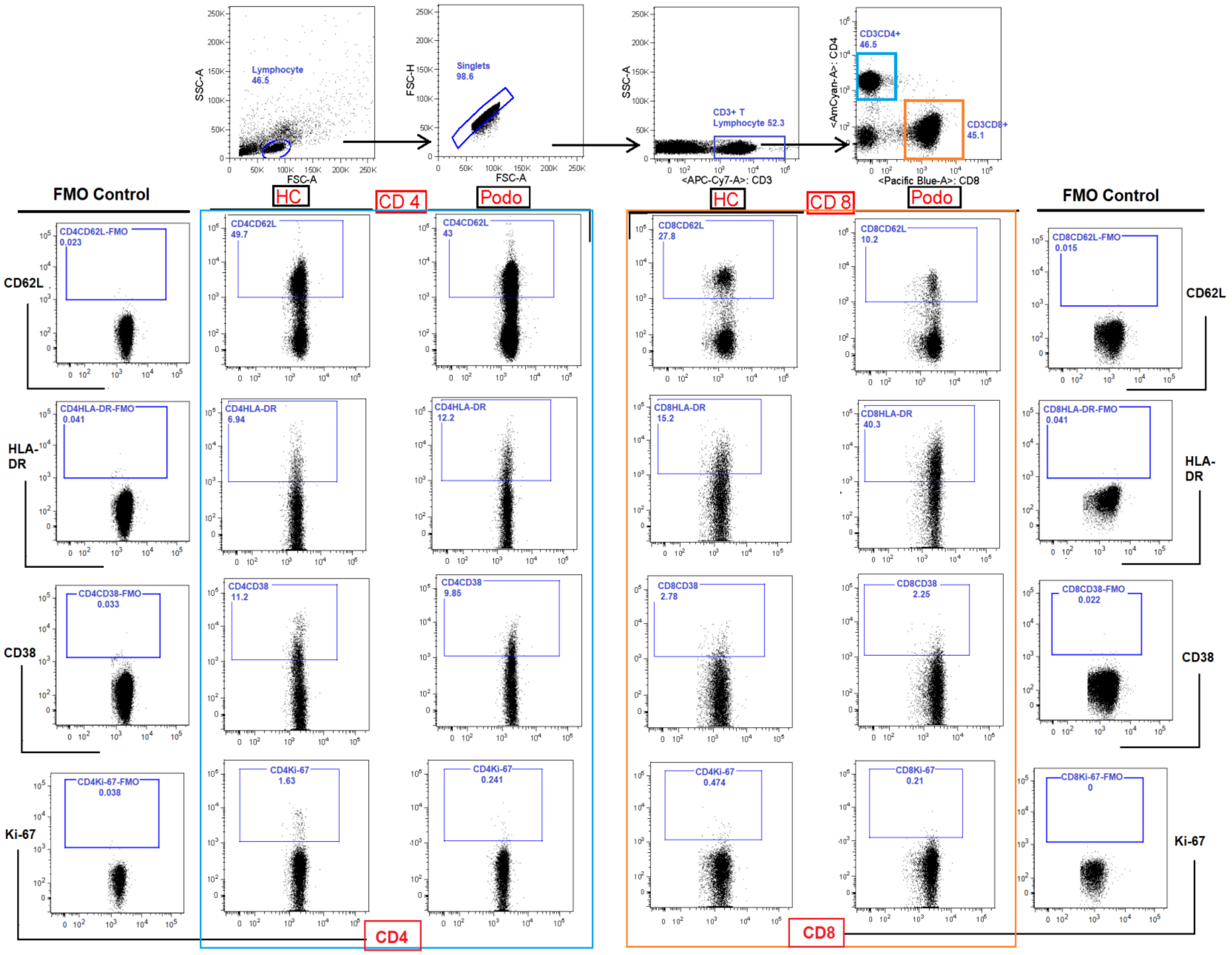

Supplementary Fig 2. Gating of T cell sub-populations from PBMCs with different biomarkers.

Hierarchical gating was used as indicated by the arrows (top row). First lymphocytes were gated according to forward and side scatter, followed by singlets gating (based on the forward scatter pulse area and height), followed by CD3 T lymphocytes, and finally CD4+CD8- and CD8+CD4- subsets. Then surface and intracellular markers were subsequently defined from gated CD4 and CD8 subpopulations. Rows two to five shows representative dot plots for one healthy control (HC) and one podoconiosis (Podo) patient depicting lymphocyte staining with CD62L, HLA-DR, CD38 and Ki-67 respectively. Column 2 shows CD4 T cells from healthy control (HC); column 3 shows CD4 T cells from a podoconiosis patient (Podo); column 4 shows CD8 T cells from a healthy control (HC); and column 5 shows CD8 T cells from podoconiosis patient (Podo). Thresholds to define cells positive for HLA-DR, CD38, CD62L and Ki-67 were based on unstained controls and florescent minus one (FMO) control, (HC, n= 44; Podo, n = 56).

Supplementary figure 3

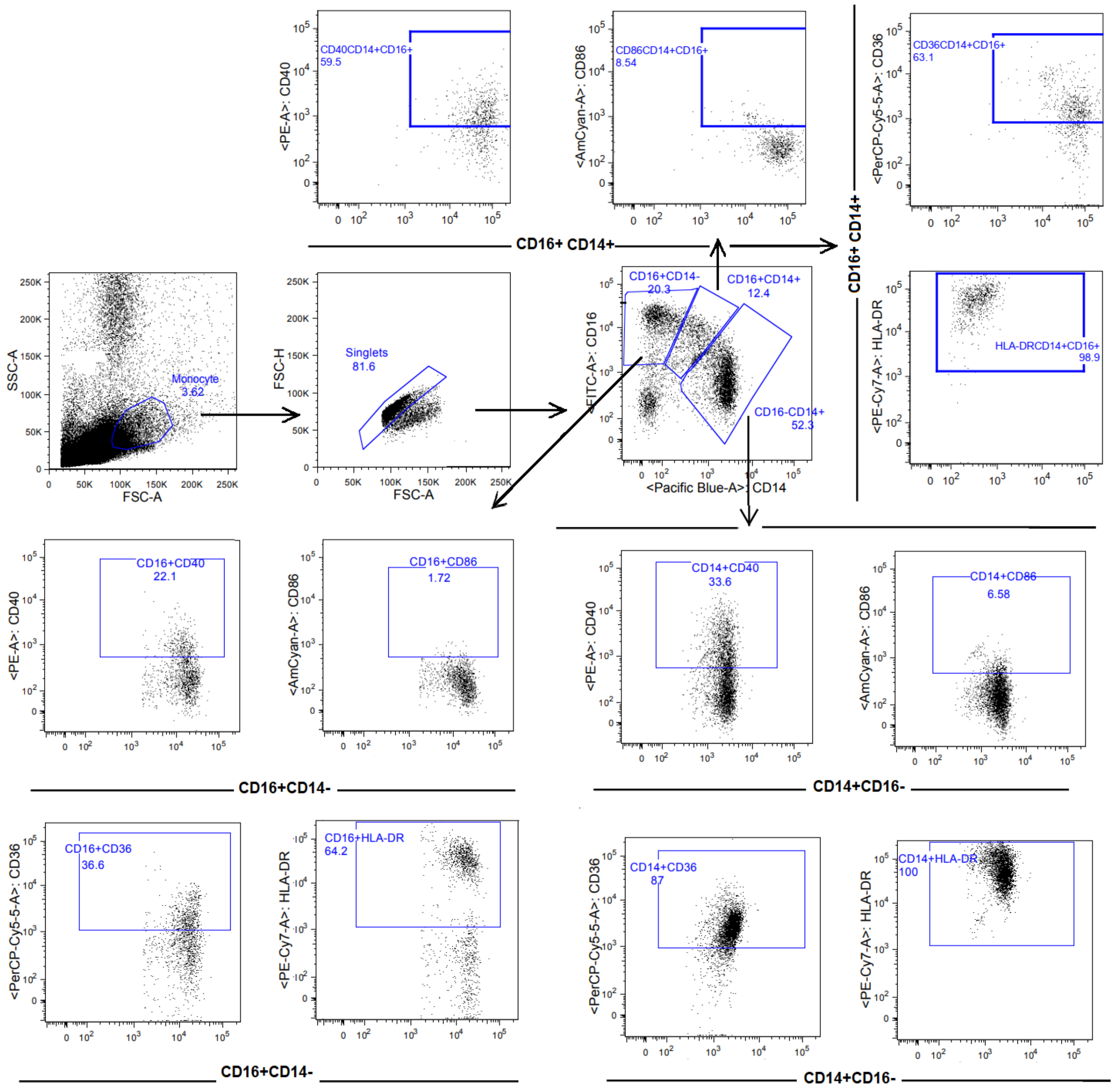

Supplementary Fig 3. Gating of monocyte subsets from PBMCs with different biomarkers.

Hierarchical gating was used as indicated by the arrows with sequential selection of monocyte areas by forward and side scatter followed by gating singlets. Then classical (CD14+CD16-), non-classical (CD16+CD14-) and intermediate (CD14+CD16+) monocyte subsets were gated based on CD14 and CD16 expression (upper row plot, left to right respectively). Finally, CD36, HLA-DR, CD40 and CD86 expression among these gated subsets was assessed. Representative dot plots from one podoconiosis patient are depicted here.

Supplementary figure 4

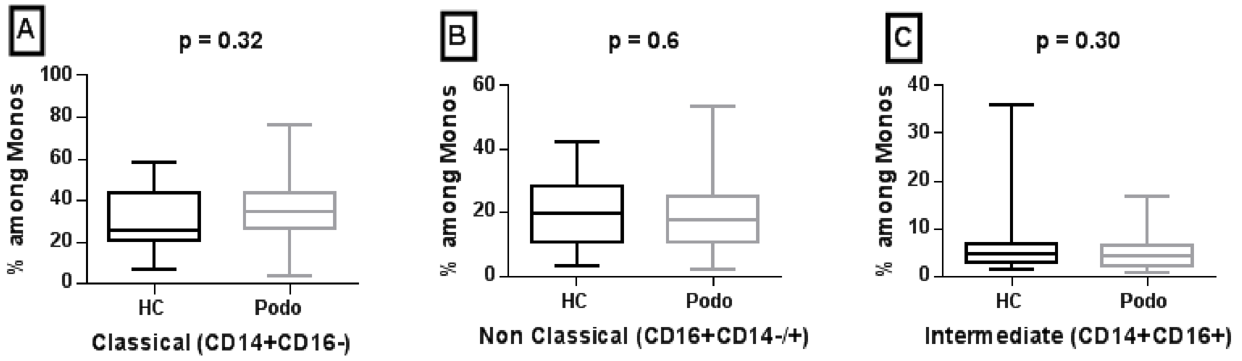

Supplementary Fig 4. Proportion of monocyte subsets in PBMC from podoconiosis patients and healthy controls.

A: Classical, B: Non-classical, and C: Intermediate. The figure shows box plots depicting median, inter-quartile range, minimum and maximum values of subset frequencies defined from PBMC from 43 podoconiosis patients and 34 healthy controls. P values were derived using the Mann Whitney U test of two sided independent t test.

Supplementary figure 5

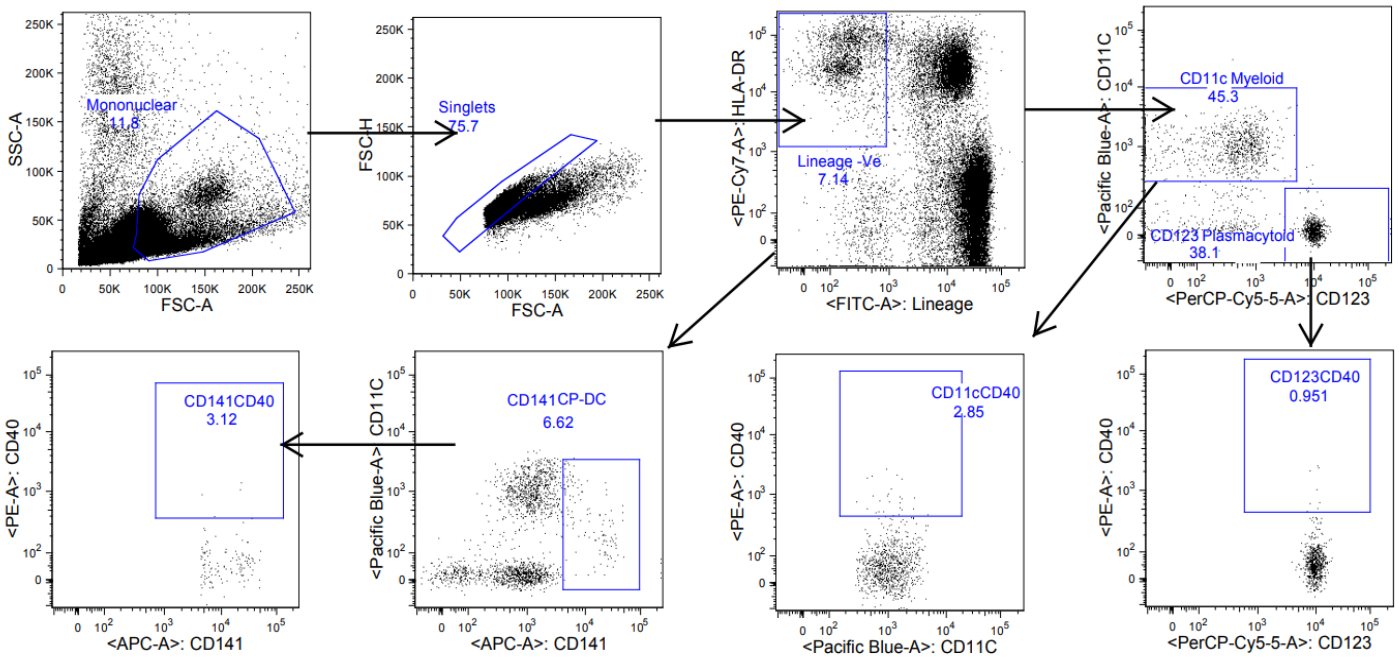

Supplementary Fig 5. Gating of dendritic cell subsets from PBMCs with different biomarkers.

Hierarchical gating was used (shown by the arrows, starting in top left-hand corner) with mononuclear cells selected by forward and side scatter, followed by singlets and DCs gated based on lineage negative and HLA-DR+ expression. Subsequently, the DC subsets were further selected by expression of CD11c, CD123 and CD141 expression into myeloid (mDC), plasmacytoid (pDC) and cross-presenting DC subsets

(cp-DC) respectively. Expression of activation markers CD40 and CD86 were further characterized for these subsets. A representative dot plot from one podoconiosis patient is depicted here.

Supplementary figure 6

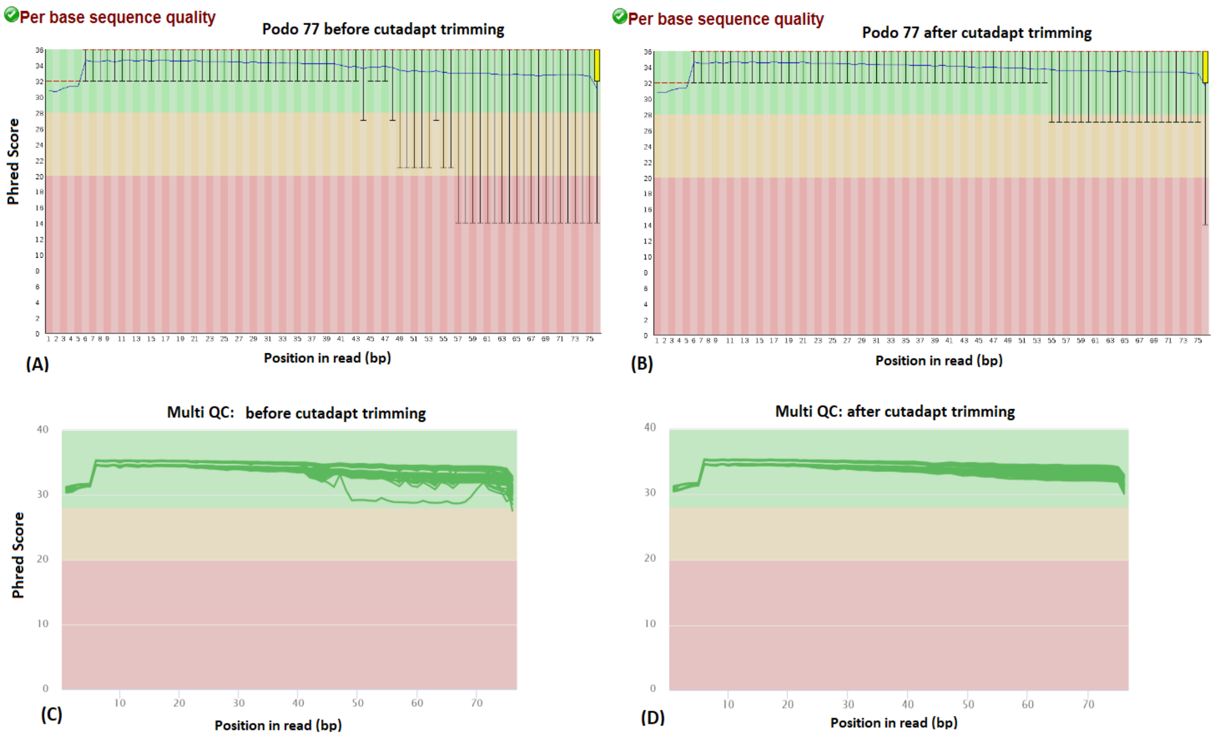

Supplementary Fig 6. Example of quality trimming of reads using cutadapt.

The quality score of a representative sample (Podo 77) is presented before (A) and after (B) quality trimming using cutadapt. Quality scores were generated with MultiQC for all samples before (C) and after (D) trimming with cutadapt. It is evident from this graph that all poor base calls were removed from subsequent pipelines. The y axis values represent Phred score where a base score of >30 is considered high quality, and the x axis values represent the position of the base in the 75 bp single end read.
